# Interaction between Endothelin-1 and Left Stellate Ganglion Activation: A Potential Mechanism of Malignant Ventricular Arrhythmia during Myocardial Ischemia

**DOI:** 10.1155/2019/6508328

**Published:** 2019-05-12

**Authors:** Zhenya Wang, Shuyan Li, Huanzhu Lai, Liping Zhou, Guannan Meng, Menglong Wang, Yanqiu Lai, Zhuo Wang, Hui Chen, Xiaoya Zhou, Hong Jiang

**Affiliations:** ^1^Department of Cardiology, Renmin Hospital of Wuhan University, Wuhan, 430060 Hubei, China; ^2^Cardiovascular Research Institute, Wuhan University, Wuhan, 430060 Hubei, China; ^3^Hubei Key Laboratory of Cardiology, Wuhan, 430060 Hubei, China; ^4^Department of Cardiology, First Hospital of Jilin University, Changchun, 130021 Jilin, China

## Abstract

Endothelin-1 (ET-1) is synthesized primarily by endothelial cells. ET-1 administration in vivo enhances the cardiac sympathetic afferent reflex and sympathetic activity. Previous studies have shown that sympathetic hyperactivity promotes malignant ventricular arrhythmia (VA). The aim of this study was to investigate whether ET-1 could activate the left stellate ganglion (LSG) and promote malignant VA. Twelve male beagle dogs who received local microinjections of saline (control, *n* = 6) and ET-1 into the LSG (*n* = 6) were included. The ventricular effective refractory period (ERP), LSG function, and LSG activity were measured at different time points. VA was continuously recorded for 1 h after left anterior descending occlusion (LADO), and LSG tissues were then collected for molecular detection. Compared to that of the control group, local ET-1 microinjection significantly decreased the ERP and increased the occurrence of VA. In addition, local microinjection of ET-1 increased the function and activity of the LSG in the normal and ischemic hearts. The expression levels of proinflammatory cytokines and the protein expression of c-fos and nerve growth factor (NGF) in the LSG were also increased. More importantly, endothelin A receptor (ETA-R) expression was found in the LSG, and its signaling was significantly activated in the ET-1 group. LSG activation induced by local ET-1 microinjection aggravates LADO-induced VA. Activated ETA-R signaling and the upregulation of proinflammatory cytokines in the LSG may be responsible for these effects.

## 1. Introduction

Malignant ventricular arrhythmia (VA) is a main cause of sudden cardiac death after acute myocardial infarction. Studies have demonstrated that cardiac sympathetic hyperactivity is a key factor in the initiation and maintenance of VA [1, 2]. Cardiac sympathetic left stellate ganglion (LSG) activity increases markedly before VA onset in an ischemia model [1], and inhibition of LSG activity effectively reduces the incidence of VA [3]. A recent clinical study also demonstrated that stellate ganglion dysfunction results in excessive and dysfunctional efferent sympathetic tone in patients with cardiomyopathy and refractory VA [4]. These findings indicate that LSG hyperactivity may be a major trigger for malignant VA. In addition, clinical studies have shown that the plasma levels of endothelin-1 (ET-1) are markedly increased in patients with sympathetic hyperactivity cardiovascular disease, such as patients with myocardial infarction [5] and hypertension [6], which suggests that ET-1 may play a key role in the regulation of sympathetic activity.

ET-1 was identified as a peptide with strong vasoconstrictive effects; it is synthesized primarily by vascular endothelial cells and by a variety of other cells, including neurons and astrocytes, and it exerts its effects through two subtypes of G-protein coupled receptor, known as endothelin A receptor (ETA-R) and endothelin B receptor [7–9]. The presence of ET-1 and ETA-R in different regions of the brain suggests that ET-1 plays a role in neuroendocrine modulation [8]. Furthermore, sympathetic neurons extend axons mainly along arteries, innervating a large variety of distinct ultimate target tissues during development [10], and the endothelins, especially ET-1, serve as vascular-derived axonal guidance cues for the development of sympathetic neurons [10, 11]. In an animal model of postinfarct ventricular tachycardia, the occurrence of arrhythmia was closely related to impaired sympathetic innervation [12]. The above studies suggest that ET-1, sympathetic nerves, and malignant VA are closely related.

Abundant ET-1 expression is found in the paraventricular nucleus (PVN) [13], which is an important integrative center in the control of the cardiac sympathetic afferent reflex (CSAR) [14]. The microinjection of ET-1 into the bilateral PVN enhances the CSAR and increases renal sympathetic nerve activity via binding to ETA-R [15]. Based on the above studies, we hypothesized that increased ET-1 in the LSG may contribute to LSG remodeling, which would result in LSG hyperactivity and subsequent malignant VA. In the present study, ET-1 was microinjected locally into the LSG, and its effects on LSG remodeling and ventricular electrophysiology were detected in a canine ischemia model.

## 2. Materials and Methods

### 2.1. Experimental Animals and Surgical Preparation

All animal experiment protocols were performed according to the National Institutes of Health guidelines and approved by the Animal Care and Use Committees of Renmin Hospital of Wuhan University. Twelve male beagle dogs with body weights of 10~12 kg were anesthetized with 3% sodium pentobarbital at an initial dose of 1 mL/kg and a maintenance dose of 2 mL/h. ECG signals and blood pressure were recorded using a computer-based lab system (Lead 7000, Jinjiang Inc., Chengdu, China) throughout the experiments.

Unilateral thoracotomy was performed at the fourth intercostal space. Acute ischemia was established by left anterior descending occlusion (LADO) for 60 minutes and confirmed by changes in the acute ST segment and T wave on surface ECG. The VA during the 60 minutes after myocardial ischemia, including ventricular tachycardia (VT, ≥3 consecutive premature ventricular beats) and ventricular fibrillation (VF), was recorded by the Lead 7000 system and numbered manually after the experiment according to Wang et al. [16].

### 2.2. Experimental Protocol

To test whether ET-1 can directly activate the LSG, we performed local microinjection of ET-1 (*n* = 6) or 0.9% saline (*n* = 6) into the LSG. LSG function and activity and the effective refractory period (ERP) were measured before and 30 min after ET-1 or saline administration. Then, LADO was performed and the VA during the 60 min after LADO was recorded. At the end of the experiment, LSG tissues were collected for molecular detection.

### 2.3. Local Microinjection of ET-1 into the LSG

A 0.1 mL volume of ET-1 (Enzo Biochem Inc., New York, USA, 0.25 mg/mL) or 0.9% saline was injected into the LSG at four points under direct visual control to ensure optimal local microinjection.

### 2.4. Measurement of the Ventricular ERP

**Figure 1:**
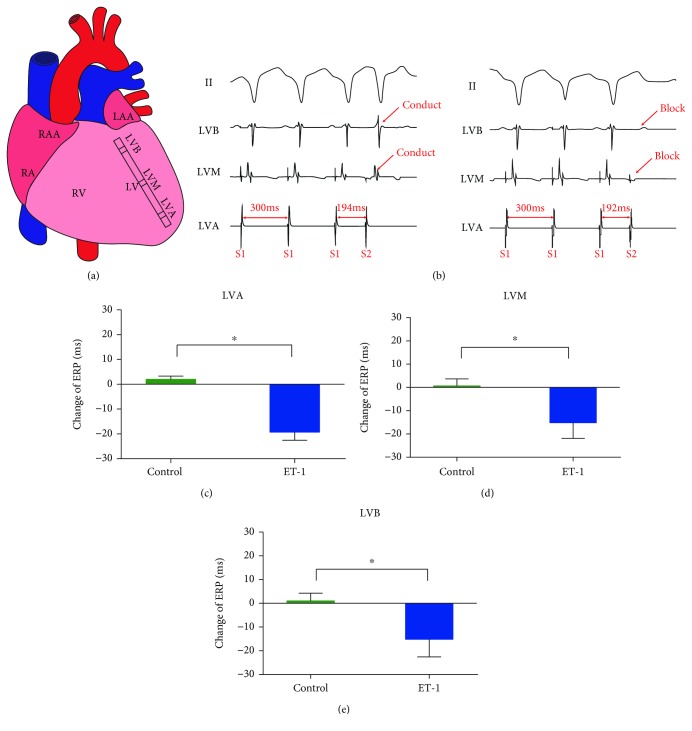
Local ET-1 microinjection increased ventricular electrophysiological instability in the normal hearts. (a) Schematic representation of the electrode position in the left ventricular free walls. (b) Representative ECG during ERP detection. (c–e) The effects of local ET-1 microinjection on ventricular ERP in the normal hearts. ^∗^*p* < 0.05. ERP: effective refractory period; LAA: left atrial appendage; RA: right atrium; RAA: right atrial appendage; RV: right ventricle; LV: left ventricle; LVA: left ventricular apex; LVB: left ventricular base; LVM: median area between the LVA and LVB.

Multielectrode catheters were sutured to the left ventricular free walls. The ventricular ERP was examined from the following three sites (Figure 1(a)): the left ventricular apex (LVA), the left ventricular base (LVB), and the median area of the left ventricle (LVM). The ERP at each site was examined by programmed stimulation comprising 8 basic stimulation conditions (S1, basic stimulus; S2, a premature stimulus; S1-S1, the interval between S1 and S1; S1-S2, the interval between S1 and S2) (S1-S1, 350 ms cycle length), followed by a premature stimulus (S2). The ERP was defined as the longest S1-S2 interval that failed to capture the ventricles. The S1-S2 interval was progressively decreased from an initial 250 ms by decrements of 10 ms and decrements of 2 ms when approaching ERP until refractoriness was achieved.

### 2.5. Evaluation of LSG Function and Neural Activity

Evaluation of LSG function and neural activity uses the method of Wang et al. [16] and Huang et al. [17], and the methods' description partly reproduces their wording. Briefly, high-frequency stimulation (HFS; 20 Hz, 0.1 ms pulse duration at different voltages) was applied to LSG using a Grass-S88 stimulator (Astro-Med, West Warwick, Rhode Island, USA). Due to the significant variation in SBP-elevating responses to HFS in each beagle dog, four incremental voltage levels (level 1 = 1 to 5 V, level 2 = 5 to 7.5 V, level 3 = 7.5 to 10 V, and level 4 = 10 to 15 V) were used for LSG stimulation. The relative change in maximal systolic blood pressure (SBP) in response to direct electrical stimulation of the LSG reflected LSG function. LSG function was evaluated at baseline and 30 min after local microinjection of ET-1 or saline.

LSG neural activity was recorded for 1 min at baseline, 30 min after local microinjection of ET-1 or saline, and 15 min after LADO. A pair of tungsten-coated microelectrodes was inserted into the fascia of the LSG, and the ground lead was connected to the chest wall to reduce noise. Neural recordings from the LSG were detected using a PowerLab data acquisition system (8/35, AD Instruments, New South Wales, Australia) and amplified using an amplifier (DP-304, Warner Instruments, Hamden, CT, USA) with bandpass filters set at 300 Hz (high-pass) to 1 kHz (low-pass) and an amplification range of 30–50 times. Neural activity, characterized by the recorded amplitude and frequency, was defined as deflections with a signal-to-noise ratio greater than 3 : 1 and was determined manually according to Wang et al. [3] and Yu et al. [18].

### 2.6. Western Blotting and Real-Time PCR

At the end of the experiment, fresh LSG tissues were excised rapidly, washed with 0.9% saline, dissected into small portions, and maintained at -80°C until use. Western blot analysis was performed to examine protein expression levels of P-PI3K, PI3K, AKT2, P-GSK3*β*, and GSK3*β* in LSG tissue. The primary antibodies used were anti-P-PI3K (Bioss, Woburn, Massachusetts, USA), anti-PI3K (Abcam Trading (Shanghai) Company, Shanghai, China), anti-AKT2 (Biorbyt, Cambridge, Cambridgeshire, United Kingdom), anti-P-GSK3*β* (Abcam Trading (Shanghai) Company, Shanghai, China), and anti-GSK3*β* (Bioss, Woburn, Massachusetts, USA). Protein expression levels were normalized to *β*-actin (CST, Danvers, MA, USA). Real-time PCR was used to quantitatively describe the mRNA expression of IL-1*β*, IL-6, and TNF-*α*. For quantification, the expression levels of mRNAs were normalized to the reference gene GAPDH.

### 2.7. Histological Staining

Hematoxylin-eosin (HE) staining was used to reveal the elementary structure of LSG and the infiltration of inflammatory cells. At the end of the experiment, the LSG tissues were excised rapidly and fixed in 4% paraformaldehyde at room temperature. Paraffin-embedded LSG tissue was cut into 5 *μ*m sections. The LSG sections were examined by light microscopy and photographed with a digital camera. Images were analyzed with Image-Pro Plus (Version 6.0) in a blinded manner.

Immunofluorescence staining was used to determine the expression and localization of ETA-R, nerve growth factor (NGF), c-fos, and tyrosine hydroxylase (TH) in the LSG. The LSG sections were incubated in PBS containing 10% fetal bovine serum for 60 min and incubated overnight at 4°C with primary antibodies, including anti-ETA-R (Enzo Biochem, New York, USA), anti-c-fos (Santa Cruz Biotechnology, Dallas, Texas, USA), anti-NGF (Abcam, Cambridge, England), and anti-TH (Abcam, Cambridge, England) antibodies. The sections were washed with PBS and incubated with the secondary antibody for 1 h at 37°C. The nuclei were stained with 4′,6-diamidino-2-phenylindole (DAPI). All images were obtained at 400x with a fluorescence microscope (Olympus DX51) and DP2-BSW software 2.2 (Olympus) and analyzed with Image-Pro Plus 6.0 (Media Cybernetics) in a blinded manner.

### 2.8. Statistical Analysis

All continuous data are expressed as the mean ± standard deviation and were analyzed by unpaired *t*-tests or two-way ANOVA. The Mann–Whitney *U* test was used to analyze the incidence of VT/VF. SPSS 19.0 and GraphPad Prism 6.0 software were used for data analysis and graphing. Differences for which *p* < 0.05 were considered statistically significant.

## 3. Results

### 3.1. Effect of ET-1 on LSG Function

**Figure 2:**
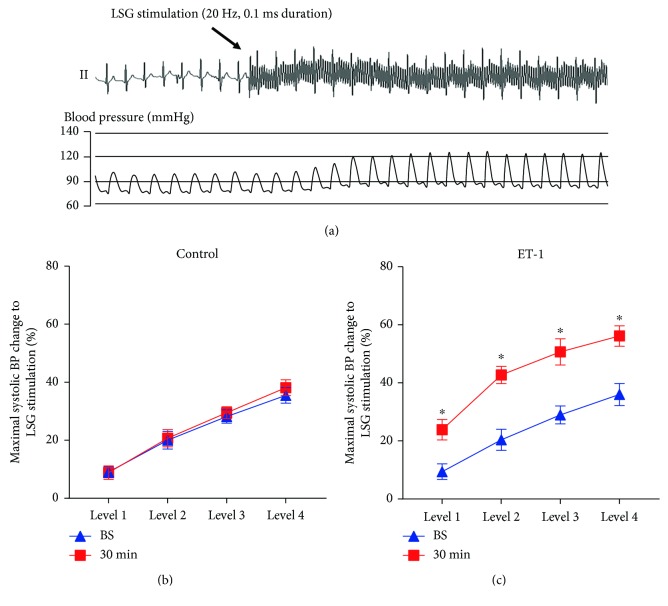
Local ET-1 microinjection increased LSG function. (a) Representative blood pressure (BP) elevation in response to LSG electrical stimulation. (b) The effects of ET-1 on LSG function in the two groups. ^∗^*p* < 0.05. BP: blood pressure.

HFS of the LSG significantly increased SBP in the control and ET-1 groups at baseline. In the control group, there were no significant differences in LSG function before or after local microinjection (Figure 2(b)). However, the LSG function 30 min after local ET-1 microinjection was significantly increased, as indicated by the increased relative change in maximal SBP in response to electrical stimulation at the same voltage level compared to the baseline (Figure 2(c)).

### 3.2. Effect of ET-1 on LSG Neural Activity

**Figure 3:**
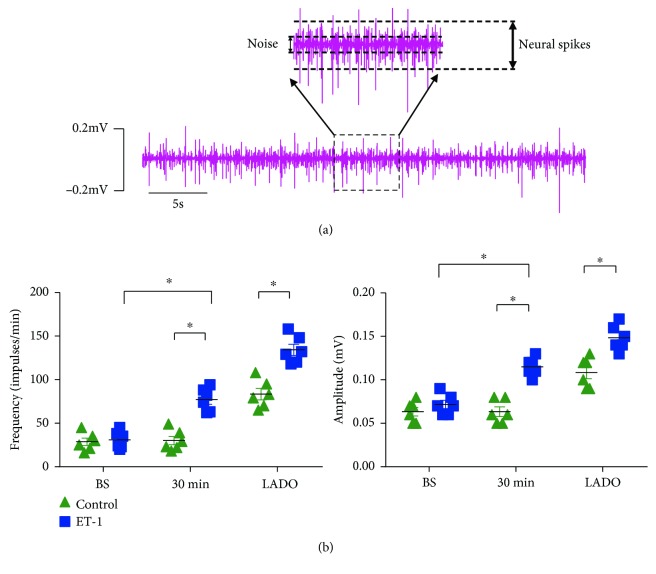
Local ET-1 microinjection increased LSG activity. (a) Representative schematic of spontaneous LSG spikes. (b) Quantification of the frequency and amplitude of LSG neural spikes. ^∗^*p* < 0.05. LADO: left anterior descending occlusion.

Figure 3(b) shows that no significant differences in LSG neural activity were observed between the two groups at baseline. Local ET-1 microinjection significantly increased the frequency and amplitude of spontaneous LSG spikes in the normal hearts, whereas no significant difference was found in the control group (Figure 3(b)). Myocardial ischemia significantly increased LSG neural activity, as shown by the increased frequency and amplitude of spontaneous LSG spikes in the myocardial ischemia group compared to those in the control group. As expected, local ET-1 microinjection further aggravated the increased LSG activity induced by myocardial ischemia (Figure 2(b)).

### 3.3. Effect of ET-1 on Ventricular ERP

Basic ventricular electrophysiology was investigated with left ventricular ERP. As shown in Figure 1, no significant differences in ERP were found after local saline microinjection. The ERP at three sites was significantly decreased after local ET-1 microinjection compared to that at baseline.

### 3.4. Effect of ET-1 on the Incidence of VT/VF in the Ischemic Hearts

**Figure 4:**
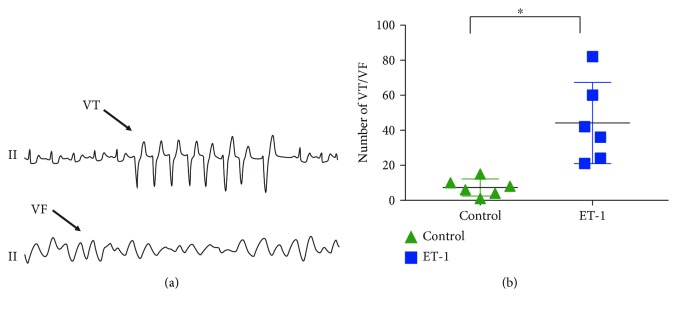
Local ET-1 microinjection increased the incidence of VT/VF in the ischemic hearts. (a) Representative ECG of ischemia-induced VT/VF. (b) The effects of local ET-1 microinjection on the incidence of VT/VF in the ischemic hearts. ^∗^*p* < 0.05. VT: ventricular tachycardia; VF: ventricular fibrillation.

Typical examples of VT/VF induced by ischemia are shown in Figure 4(a). Compared to that in the control group, the number of VT and VF episodes was significantly increased in the ET-1 group (Figure 4(b)).

### 3.5. Effect of ET-1 on the Inflammatory Response in the LSG

**Figure 5:**
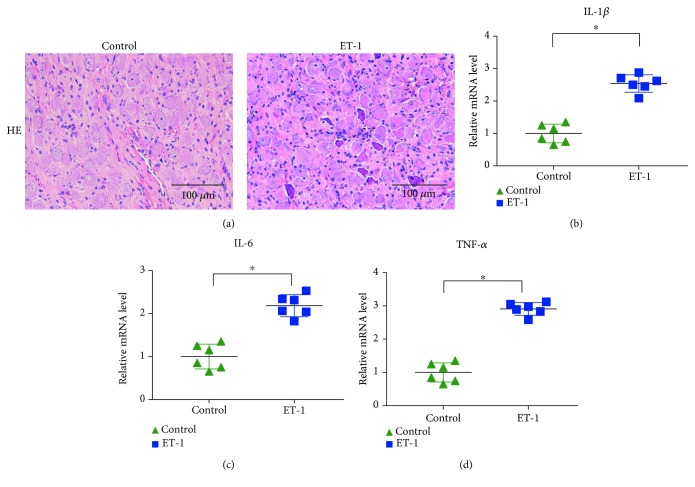
Local ET-1 microinjection increased the inflammatory response in the LSG. (a) Representative image of HE staining. (b–d) The effects of local ET-1 microinjection on the expression levels of IL-1*β*, IL-6, and TNF-*α*. ^∗^*p* < 0.05.

The expression of proinflammatory cytokines was detected to reveal the severity of inflammatory cell infiltration around the neurons in the LSG (Figure 5(a)). The staining showed a low level of inflammatory cell infiltration in the LSG in the control group, whereas aggravated inflammatory cell infiltration was found in the ET-1 group. Additionally, the mRNA levels of the proinflammatory cytokines IL-1*β*, IL-6, and TNF-*α* were significantly increased in the ET-1 group.

### 3.6. Effect of ET-1 on the Expression of c-fos and NGF in the LSG

**Figure 6:**
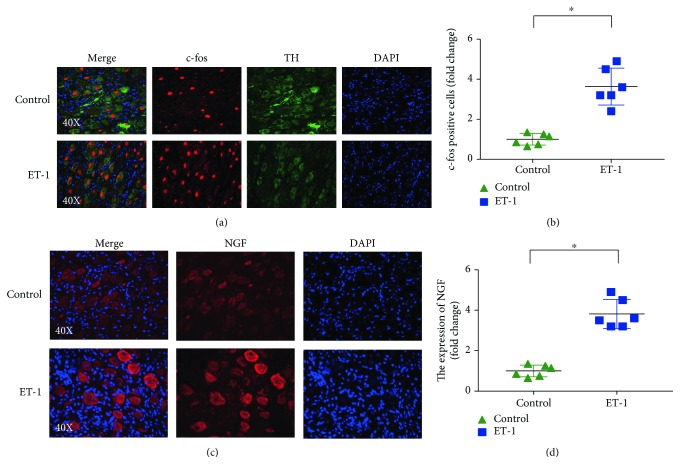
Local ET-1 microinjection increased the expression of c-fos and NGF in the LSG. (a) Double staining of c-fos (red) and TH (green) to indicate the activation of sympathetic neurons. (b) Quantitative analysis of the expression of c-fos in the LSG in different groups. (c) Representative image of simple NGF staining. (d) Quantitative analysis of the expression of NGF in the LSG in different groups. ^∗^*p* < 0.05. TH: tyrosine hydroxylase; NGF: nerve growth factor.

Immunofluorescence staining of the LSG in the ET-1 group showed that increased expression levels of c-fos in the LSG were primarily localized to sympathetic neurons that stained with TH, which is a marker for sympathetic neurons. Quantitative data indicated that the number of c-fos-positive sympathetic neurons in the ET-1 group was also dramatically increased (Figure 6(b)). Compared to those in the control group, the expression levels of NGF were significantly increased in the ET-1 group (Figure 6(d)).

### 3.7. ET-1 Activates the PI3K/Akt/GSK-3*β* Pathway in the LSG

**Figure 7:**
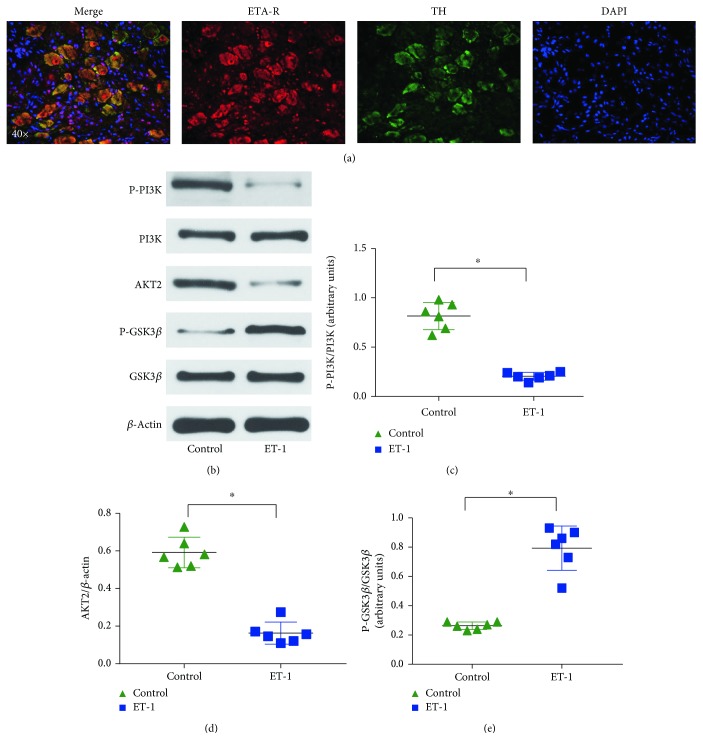
Local ET-1 microinjection activates the PI3K/Akt/GSK-3*β* pathway in the LSG. (a) Representative images showing double-immunofluorescence staining for ETA-R (red) and TH (green) in the LSG. (b) Representative images of the Western blots. (c–e) Quantitative analyses of the Western blot results showed that the ETA-R and PI3K/Akt/GSK-3*β* pathways were activated in the LSG. ^∗^*p* < 0.05. ETA-R: ETA receptor; TH: tyrosine hydroxylase.

Based on the immunofluorescence results concerning ETA-R+ TH+ double-positive cells in the LSG, we revealed ETA-R expression in the cardiac autonomic nervous system (Figure 7(a)). The protein expression of P-PI3K, PI3K, AKT2, P-GSK3*β*, and GSK3*β* in the LSG was evaluated (Figure 7(b)), and the results indicated that compared to the control treatment, ET-1 microlocal microinjection significantly decreased the activation of PI3K and Akt and stimulated the phosphorylation of GSK-3*β* in the LSG, which induced the activation of the PI3K/Akt/GSK-3*β* pathway via ET-1 binding to the ETA-R as a potential mechanism for LSG hyperactivity.

## 4. Discussion

### 4.1. Major Findings

In the present study, we evaluated the effects of ET-1 on LSG neural remodeling, the inflammatory response, and the incidence of malignant VA in a canine model using beagles. The study results showed the following: (1) the local microinjection of ET-1 into the LSG reduced left ventricular ERP, which contributed to the increased incidence of VA induced by myocardial ischemia; (2) the local microinjection of ET-1 into the LSG resulted in sympathetic hyperactivity; (3) the local microinjection of ET-1 into the LSG increased the expression of c-fos and NGF in the LSG, which contributed to increased sympathetic activity; (4) the local microinjection of ET-1 into the LSG increased the expression of proinflammatory cytokines; and (5) the local microinjection of ET-1 into the LSG significantly decreased the activation of PI3K and Akt and stimulated the phosphorylation of GSK-3*β* in the LSG. These results indicate that ET-1 contributes to LSG hyperactivity, possibly through activating the ETA-R signaling pathway directly and upregulating proinflammatory cytokines indirectly, which increases the incidence of malignant VA.

### 4.2. ET-1 Activated the LSG to Induce Malignant VA

Clinical studies have shown that the plasma levels of ET-1 are markedly increased in patients with acute coronary syndrome induced by emotional stress compared to those in similar patients without apparent sympathetic activation [19]. Furthermore, low-dose ET-1 administered via the intrapericardial [20] or intracoronary [21] route resulted in VA in vivo in large animal models. The above research suggests that there are strong relationships among ET-1, the sympathetic system, and the resultant effects on arrhythmogenesis.

A previous study demonstrated that impaired cardiac autonomic control contributes to increased inducibility of malignant VA [22] and that sympathetic hyperactivity results in the reduction of ventricular ERP and APD [23, 24]. In the present study, we found that ET-1 application decreased ventricular ERP, which demonstrated that electrophysiologic instability may be increased in the ET-1 group. In addition, our results showed increased LSG function and neural activity in the ET-1 group, which further verified that local ET-1 microinjection into the LSG resulted in sympathetic hyperactivity.

Consistent with these findings, our results showed that local ET-1 microinjection into the LSG could contribute to cardiac sympathetic hyperactivity and thus increase the occurrence of malignant VA.

### 4.3. Potential Mechanisms

**Figure 8:**
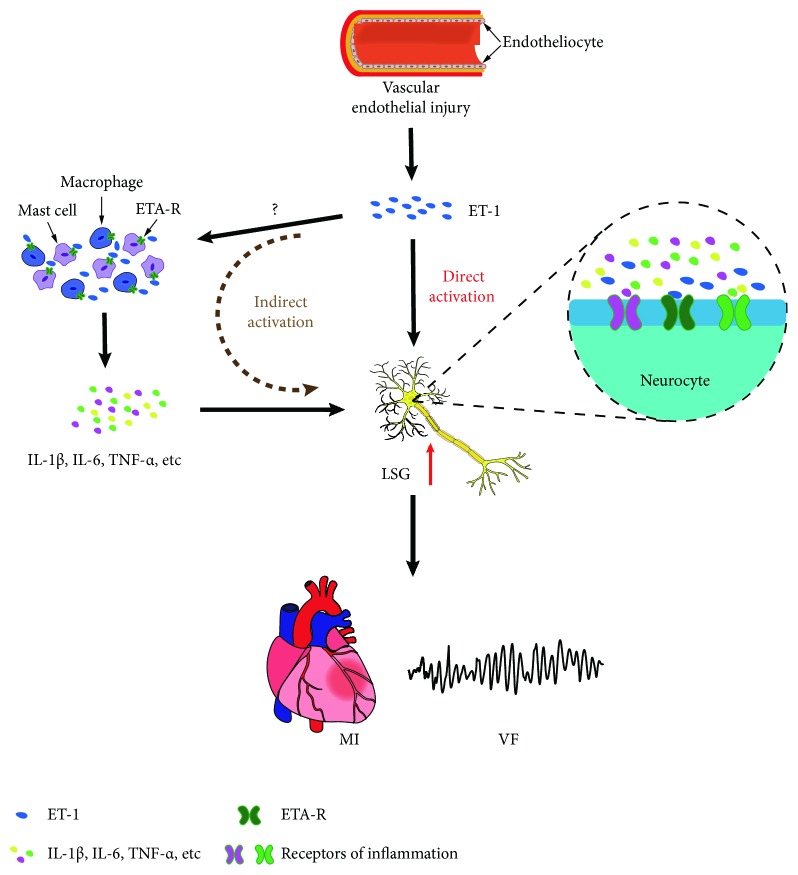
Schematic diagram depicting the potential role of ET-1 in the LSG and the aggravation of VA. ET-1 is mainly synthesized by vascular endothelial cells and contributes to cardiac sympathetic hyperactivity, aggravating LADO-induced VA. The underlying mechanisms may be correlated directly with activated ETA-R signaling in the LSG or correlated indirectly with an increased inflammatory response.

It has been demonstrated that sympathetic ETA-R is required for pathological cardiac remodeling and disturbed sympathetic nerve function [25]. Previous studies have shown that ETA-R exists in the sympathetic nerve terminals of the heart [26]. In this study, our results showed the presence of ETA-R on the sympathetic neurons in the LSG (Figure 7), indicating that ET-1 may be involved in several functions. Additionally, ETA-R activates multiple signaling pathways, which include the oxidative stress, the extracellular signal-regulated kinase 1 and 2 (ERK1/2) pathway, the phosphoinositide 3-kinase (PI3K) pathway, and the glycogen synthase kinase- (GSK-) 3*β* signaling pathway [27, 28]. In particular, an ETA-R antagonist attenuated sympathetic hyperinnervation and inhibition of the PI3K/Akt/GSK-3*β* signaling pathway, which indicated that the PI3K/Akt/GSK-3*β* signaling pathway may play an important role in the activation of sympathetic neurons [28]. In this study, we showed that local microinjection of ET-1 into the LSG activated the PI3K/Akt/GSK-3*β* signaling pathway and that the activated ETA-R signaling pathway may play a key role in the direct LSG activation induced by ET-1 (Figure 8).

Inflammation has been demonstrated to regulate sympathetic activity. To investigate the mechanisms that underlie the regulation of sympathetic activity by ET-1, we also tested the levels of proinflammatory cytokines that are known to be capable of increasing sympathetic nerve activity, such as TNF-*α*, IL-1*β*, and IL-6, in LSG tissues [29, 30]. Our previous studies have shown increased IL-1*β* [29] and leptin [30] levels in the LSG, which was confirmed by the proinflammatory effects of mast cells (MCs) and macrophages; these factors could significantly upregulate sympathetic activity, and these effects were reversed by an IL-1 receptor antagonist and MC stabilizer, resulting in MC inactivation [31]. In addition, previous studies have confirmed that ET-1 and ETA-R also play a role in immunoregulation [32]. MCs and macrophages are important immune cells that express ETA-R [33, 34]; MCs and macrophages drive inflammation, and their inflammatory response can be modulated by ET-1 [34, 35], which indicates that MCs and macrophages may be potential mechanisms for the proinflammatory effect of ET-1. Thus, the above studies suggest that ET-1 increases proinflammatory cytokines, possibly by activating MCs and macrophages, which may provide another means by which local ET-1 microinjection indirectly results in LSG hyperactivity (Figure 8).

### 4.4. Clinical Significance

ET-1 is widely distributed in the majority of organs and tissues and is involved in physiological regulation. ET-1 is closely related to the occurrence of various cardiovascular diseases, such as myocardial infarction, hypertension, and heart failure. The results of this study provide more evidence that ET-1 is a reliable risk marker and a potential therapeutic target for ischemic VA.

### 4.5. Study Limitations

This study involved an investigation of acute effects, and the long-term effect of increased ET-1 in the LSG on sympathetic neural remodeling and ischemic VA remains to be elucidated.

## 5. Conclusions

In the present study, we found that local microinjection of ET-1 into the LSG resulted in LSG neuronal remodeling and aggravated ischemia-induced VA. Activation of the ETA-R signaling pathway and inflammation-mediated sympathetic neural remodeling could be a novel mechanism and therapeutic target for ischemic VA.

## Data Availability

The data used to support the findings of this study are available from the corresponding authors upon request.
